# Selective Cytotoxicity in Chronic Myeloid Leukemia (K-562) Cells Induced by 532 nm LASER Irradiation Without Exogenous Photosensitizers

**DOI:** 10.3390/biomedicines13112649

**Published:** 2025-10-29

**Authors:** Danielle Viviana Ochoa-Arbeláez, Efraín Solarte-Rodríguez, Yamil Liscano

**Affiliations:** 1 Grupo de Investigación Cuidado de la Salud, Facultad de Salud, Universidad Santiago de Cali, Cali 760035, Colombia; 2 Grupo de Investigación de Farmacología, Facultad de Salud, Universidad del Valle, Cali 760043, Colombia; 3 Grupo de Investigación de Óptica Cuántica, Facultad de Ciencias Naturales y Exactas, Universidad del Valle, Cali 760043, Colombia; 4 Grupo de Investigación en Salud Integral (GISI), Facultad de Salud, Universidad Santiago de Cali, Cali 760035, Colombia; yamil.liscano00@usc.edu.co

**Keywords:** Chronic Myeloid Leukemia, K-562, biophotonics, LASER Therapy, cytotoxicity, Direct Light Stimulation

## Abstract

**Background and Objectives:** The treatment of Chronic Myeloid Leukemia (CML) faces challenges such as resistance to Tyrosine Kinase Inhibitors (TKIs), necessitating new adjuvant therapies. This study aimed to evaluate the cytotoxic effect of direct, photosensitizer-free irradiation with LASER and LED light on the CML cell line K-562, hypothesizing that LASER light at a specific wavelength would be selectively effective. This work serves as a foundational in vitro study to establish the basis for a potential ex vivo therapeutic strategy. **Methods:** The human CML cell line K-562 was irradiated with LASER (405, 532, 629 nm) and LED (457, 517, 630 nm) sources at energy doses from 1 to 10 J/cm^2^. Cell viability was assessed 24 h post-irradiation using Trypan Blue exclusion, the MTT assay, and biophysical changes in the cell absorbance spectrum. **Results:** Irradiation with a 532 nm LASER was the only condition that induced massive, statistically significant, and dose-dependent cytotoxicity, reaching up to 67.8% cell death at 10 J/cm^2^ (*p* < 0.05). In contrast, other LASER wavelengths and all tested LED wavelengths failed to produce a significant cytotoxic effect. The superiority of the LASER over the LED of a similar wavelength highlights the critical role of the physical properties of light. **Conclusions:** Direct, photosensitizer-free irradiation with 532 nm LASER light is a potent and selective method for inducing cytotoxicity in K-562 cells in vitro. This effect is critically dependent on both the specific wavelength and the optical properties of the light source. These findings establish a solid foundation for the development of new ex vivo adjuvant therapies, such as extracorporeal photopheresis, for CML, pending further validation of its mechanism and selectivity.

## 1. Introduction

CML is a myeloproliferative hematological neoplasm that accounts for 15–20% of all leukemia cases in adults. The development of TKIs has radically transformed patient prognosis, achieving 5-year survival rates of up to 85%. However, the efficacy of these drugs is compromised by the emergence of resistance, inadequate responses, adverse effects impacting adherence, and disease recurrence, which underscores the imperative need to develop new coadjuvant therapeutic strategies [1,2,3,4,5].

Second-line therapies, including chemotherapy, bone marrow transplantation, and immunotherapy, while effective in certain scenarios, pose significant challenges. These treatments are associated with high systemic toxicity, a risk of severe immunosuppression, and the complexity of finding compatible donors, factors that limit their applicability and affect the patient’s quality of life. This therapeutic landscape highlights the importance of investigating approaches with a greater selectivity profile, capable of targeting malignant cells while minimizing damage to surrounding healthy tissue [2,6,7].

In this context, biophotonics, the application of light in biological systems, emerges as a promising field for the development of innovative oncological therapies. One of its modalities, Direct Light Stimulation (DLS), is based on the ability of light to induce a cellular response without the mediation of an exogenous photosensitizer. The principle of this technique lies in the fact that cancer cells, due to their metabolic and molecular alterations, may possess endogenous chromophores that absorb light at specific wavelengths, triggering signaling cascades that can lead to cell death. While this principle is often explored for biostimulation (photobiomodulation), its potential to be harnessed for targeted cytotoxicity by exciting native chromophores remains largely under-investigated in hematological cancers. This direct interaction offers the potential for a highly selective therapy with a superior safety profile compared to conventional treatments [8,9,10].

To evaluate this hypothesis, two types of light sources with distinct physical properties were employed: LASER (Light Amplification by Stimulated Emission of Radiation) and LED (Light Emitting Diode). LASER is characterized by its high coherence, directionality, and monochromaticity, properties that allow for a highly concentrated and specific energy delivery. In contrast, LED light is non-coherent and exhibits greater angular divergence. The comparison between both sources is crucial to determine whether the physical properties of light, beyond just the wavelength, play a decisive role in biological efficacy [11,12].

Although light-based therapies, such as Photodynamic Therapy (PDT), have been explored in oncology, the vast majority rely on external photosensitizing agents. There is a notable scarcity of studies that systematically investigate the cytotoxic potential of DLS in hematological cancers. Specifically, there is a lack of research identifying optimal wavelengths and, crucially, determining how the physical properties of the light source (such as coherence) influence the biological outcome. This knowledge gap represents an opportunity to develop a new non-pharmacological therapeutic modality [8,13,14].

Based on these considerations, we hypothesized that direct optical irradiation at a specific wavelength could induce a selective cytotoxic effect in CML cells and that the unique properties of LASER would make it more effective than LED. Therefore, the primary objective of this preliminary study was to evaluate and quantify the effect of irradiation with LASER and LED light sources at different visible spectrum wavelengths on the viability of the CML cell line K-562. This work serves as an essential in vitro proof-of-concept to establish the foundational parameters for a potential adjuvant therapy, envisioned for ex vivo applications such as Extracorporeal Photopheresis (ECP).

## 2. Materials and Methods

### 2.1. Cell Line and Culture Conditions

The human CML cell line K-562 (ATCC^®^ CCL-243™, Manassas, VA, USA), characterized by the presence of the Philadelphia chromosome (BCR-ABL1), was used. The cells were cultured in suspension in Iscove’s Modified Dulbecco’s Medium (IMDM; Gibco, Grand Island, NY, USA), supplemented with 8% Fetal Bovine Serum (Gibco, Grand Island, NY, USA) and 1% Penicillin–Streptomycin antibiotic mixture (Gibco, Grand Island, NY, USA). Cultures were maintained in a humidified incubator at 37 °C with a 5% CO_2_ atmosphere. For all experiments, cells were in the exponential growth phase and were seeded at a density of 5 × 10^5^ cells/mL.

### 2.2. Spectroscopic Characterization and Definition of the Optical Therapeutic Window (OTW)

Prior to irradiation experiments, a comprehensive spectroscopic characterization of the K-562 cell suspension was performed to identify regions of maximum light–cell interaction. Using an optical setup with a halogen light source and a spectrophotometer, the Absorbance, Reflectance, and Transmittance spectra were measured in the 400–750 nm range. Additionally, Fluorescence spectra were measured by exciting the sample with a blue LASER (405 nm). The analysis of these spectra allowed for the identification of wavelengths where light absorption by the cells was highest. This region of high interaction, termed the OTW, guided the selection of wavelengths (blue, green, and red) for the cytotoxicity experiments [15,16,17].

### 2.3. Optical Setup and Irradiation Protocol

**Figure 1:**
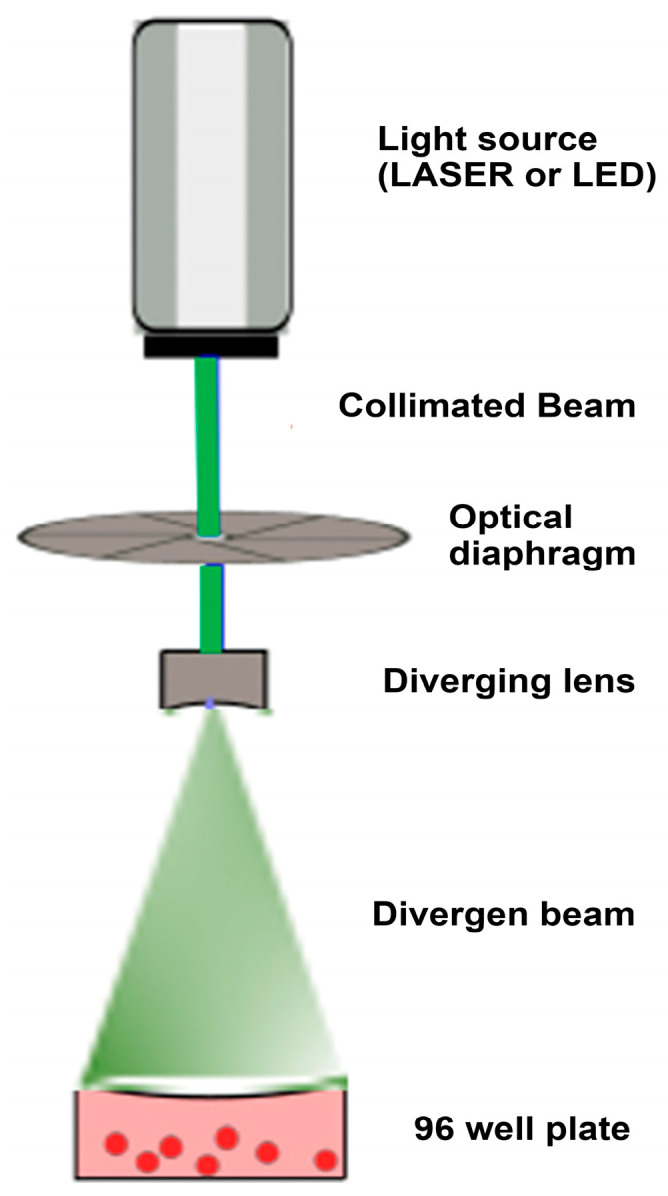
Schematic of the Optical Irradiation Protocol. The diagram illustrates the experimental setup used for the direct irradiation of K-562 cell cultures. Light emitted from the source (LASER/LED) is first collimated and then expanded by a diverging lens. This ensures that the expanded beam provides uniform and homogeneous energy coverage across the surface of the well within the 96-well plate containing the cell suspension.

A custom optical irradiation system was designed and built to ensure precise and homogeneous light delivery [15]. The experimental setup is depicted in Figure 1. Two types of light sources were used: low-power LASER diodes (405 nm, 532 nm, 629 nm) and high-intensity Light-Emitting Diodes (LEDs) (457 nm, 517 nm, 630 nm). The light beam from each source was directed vertically onto the culture plate and expanded using a diverging lens to ensure that the irradiation area completely covered the surface of each well, guaranteeing a uniform energy distribution. The distance from the final optical element (diverging lens) to the surface of the cell culture medium was fixed at 10 cm for all experiments.

The irradiance (power per unit area, in mW/cm^2^) was measured at the cell plane using a calibrated photometer with a silicon sensor (Thorlabs, Newton, NJ, USA). The exposure time was adjusted for each source to deliver total energy doses of 1, 3, 5, and 10 J/cm^2^. Given the different output powers of the sources, exposure times ranged from approximately 2 min for the highest dose with the most powerful source to over 30 min for the lowest dose with the least powerful source. All manipulations and irradiation procedures were performed under strict aseptic conditions inside a laminar flow cabinet to prevent contamination in a cell culture laboratory.

### 2.4. Experimental Design

A full factorial experimental design was implemented to evaluate the effect of three variables: light source (LASER vs. LED), wavelength (blue, green, red), and energy dose (1, 3, 5, 10 J/cm^2^). Each of the 24 treatment combinations was performed in triplicate to ensure the reproducibility of the results. Non-irradiated controls were included in each experiment to establish baseline viability.

By adjusting the optical irradiation parameters, such as the light source used, the wavelength, and the irradiation dose, which, in turn, depended on the power, the spatial distribution of the light incident on the sample, the exposure time of the cells to the light photons, and the frequency with which irradiation was carried out on the cell cultures, the optical irradiation protocol was defined, and its effect on cultured leukemic cells was studied.

To avoid contamination of the cell cultures, each seeded cell line was irradiated inside a laminar flow isolation hood with a UV lamp, following all the safety and aseptic protocols required for cell culture.

To focus the light emitted by the light sources used onto the effective area of the culture dish, a converging lens was used between the source and the sample to long of 16cm. Based on the characteristics of this lens, the source’s characteristic spot diameter could be enlarged so that the light intensity incident on the sample completely covered the seeded area in the culture dish.

Once the optimal distance between the source and the objective was determined, the intensity with which each light source hit the cell cultures was measured. This was achieved using a power meter with a silicon sensor.

Irradiation times and frequencies were calculated taking into account the light power supplied by the light source in the area of cell incidence and energy densities (light doses) between 1 and 10 (J/cm^2^) based on studies conducted by the Quantum Optics Research Group at the Universidad del Valle on other cell lines [15].

The cells were placed in a polystyrene culture cell measuring 35 × 10 mm, with a total area of 4.906 cm^2^, under optical irradiation conditions dependent on: (a) the light source used, (b) the wavelength, (c) the source power, (d) the irradiation time, and (e) the light dose; this allowed the study to evaluate the quantifiable effect of ELD. The optical experiments were carried out in triplicate to ensure reproducibility of the experimental optical conditions

### 2.5. Quantification of Cytotoxicity and Cell Viability

Cell death was evaluated 24 h after irradiation using three complementary methodologies to ensure the robustness of the findings:

Trypan Blue Exclusion Assay (Direct Count): An aliquot of the cell suspension was mixed with a 0.4% Trypan Blue solution. The principle of this assay is based on the fact that viable cells, with intact membranes, exclude the dye, whereas non-viable cells incorporate it. Stained (dead) and unstained (alive) cells were counted using a Neubauer chamber under an inverted phase-contrast microscope (Model CKX53, Olympus, Tokyo, Japan). The percentage of cell death was calculated relative to the total cell count.

Metabolic Activity Assay (MTT): Cell viability was determined using the colorimetric MTT (3-(4,5-dimethylthiazol-2-yl)-2,5-diphenyltetrazolium bromide) assay according to Equation (1).

Equation (1): Formula to determine cell viability:

(1) $$\%Viability=\;\frac{final\;cells}{Initial\;cells}\;*\;100$$

The mitochondrial dehydrogenase enzymes of metabolically active cells reduce the yellow Tetrazolium salt MTT to purple Formazan crystals. After incubation with the MTT reagent, the Formazan crystals were solubilized, and the absorbance of the resulting solution was measured at 570 nm in a ELISA microplate reader (Model iMark™, Bio-Rad Laboratories, Hercules, CA, USA), according to the Merck protocol [18]. The color intensity is directly proportional to the number of viable cells.

Spectroscopic Analysis: The absorbance spectra of the cell suspensions were characterized in the 400–750 nm range before and after irradiation. A protocol was developed to correlate the decrease in the area under the absorbance spectrum curve with the reduction in the viable cell population, providing a biophysical quantification method.

### 2.6. Processing Scientific Images

The micrographs were captured with the aforementioned inverted microscope equipped with a digital camera (SC50, Olympus, Tokyo, Japan). The density of live cells before and after optical irradiation treatment was obtained by extracting information from the micrographs. A 3D surface plot was generated, showing a greater number of peaks in the area corresponding to non-irradiated cells compared to irradiated cells. The results demonstrate that the laser successfully induced controlled cell death in K562 cells. All image analyses were performed using ImageJ software (version 1.53k, National Institutes of Health, Bethesda, MD, USA), utilizing the built-in 3D Surface Plot plugin.

### 2.7. Statistical Analysis

Data are presented as the mean ± standard deviation of three independent experiments with replicates, ensuring the reproducibility of the analysis. The normality of the data distribution was assessed using the Shapiro–Wilk test. Since the data did not follow a normal distribution, the non-parametric Kruskal–Wallis test was used to compare the medians between the different treatment groups. A *p*-value < 0.05 was considered statistically significant. All analyses were performed using STATA v.14 software (StataCorp, College Station, TX, USA).

### 2.8. Ethical Considerations

This study was conducted in accordance with institutional and national ethical guidelines. According to the official statement from the Health Research Ethics Committee of the Faculty of Health at Universidad del Valle, dated 17 January 2024, in Santiago de Cali, ethical review and approval were not required for this research proposal. The committee certified that the study, “EFFECTS OF OPTICAL IRRADIATION WITH LASER AND LED LIGHT SOURCES ON LEUKEMIA CELL CULTURES” (Code: NA 001-024, 17 January 2024), exclusively uses commercial cell lines and does not involve human subjects, either through direct participation or the use of their information.

## 3. Results

### 3.1. The Intrinsic Optical Properties of K-562 Cells Reveal a Therapeutic Window in the Visible Spectrum

**Figure 2:**
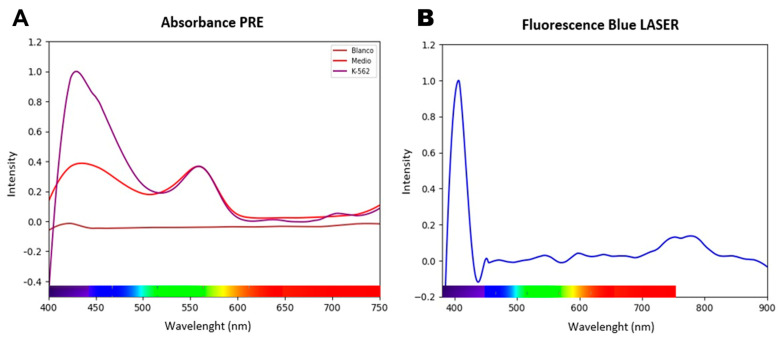
Spectroscopic characterization of the K-562 cell line for the definition of the Optical Therapeutic Window. (**A**) shows the absorbance spectrum, where K-562 cells (purple line) exhibit distinctive absorption peaks in the blue (~430 nm) and green (~560 nm) regions compared to the culture medium alone (red line), indicating the presence of endogenous chromophores. (**B**) presents the emission spectrum of the cells when excited with a 405 nm LASER; in addition to the sharp peak corresponding to scattered excitation light, a broad band of resulting fluorescence is observed in the near-infrared region (720–810 nm). Together, these spectra confirm the ability of cellular components to absorb and re-emit light energy, establishing the basis for selecting therapeutic wavelengths.

To substantiate the selection of wavelengths with therapeutic potential, an exhaustive mapping of the optical properties of the K-562 cell line was conducted. The analysis of the absorbance spectra was particularly revealing, identifying two regions of preferential interaction in the visible spectrum, as detailed in Figure 2A. The first and most pronounced absorption band was located in the blue region, with a maximum around 430 nm, while a second, smaller but clearly defined band was observed in the green region, centered at approximately 560 nm. These high-absorption regions suggest the presence of endogenous chromophores capable of initiating a photochemical cascade. This characterization was complemented by fluorescence studies, which showed that exciting the cells with a 405 nm LASER induced detectable endogenous fluorescence emission in the near-infrared (720–810 nm), as seen in Figure 2B. Collectively, these spectroscopic data defined an OTW and established the blue and green wavelengths as the most logical candidates for cytotoxicity experiments.

### 3.2. Selective Irradiation with a 532 nm LASER Induces Significant and Dose-Dependent Cytotoxicity

**Figure 3:**
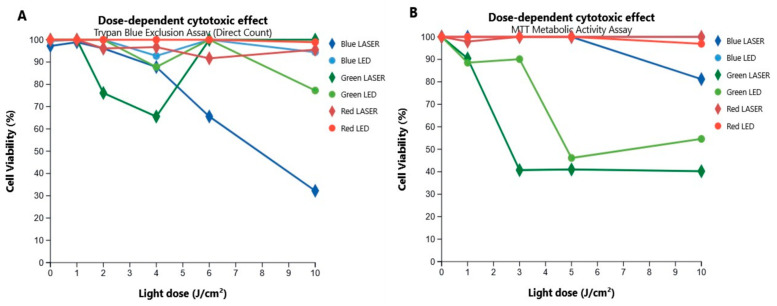
Dose-dependent effect of LASER and LED irradiation on the viability of K-562 cells. The figure shows the percentage of viable K-562 cells 24 h after irradiation, quantified by two complementary methodologies. (**A**) Cell viability as measured by the Trypan Blue exclusion direct count assay. (**B**) Cell viability as determined by the MTT metabolic activity assay. In both assays, only the 532 nm (green) LASER induced a significant, dose-dependent reduction in cell viability, while other wavelengths and all LED sources showed minimal effect. Data points represent the mean of three independent experiments. Circles denote LASER sources and diamonds denote LED sources for each corresponding color (Blue: ~405/457 nm; Green: ~532/517 nm; Red: ~629/630 nm).

The systematic evaluation of the irradiation effect revealed an extraordinarily specific cytotoxic response to the wavelength, optical irradiation conditions, and light source. Of all conditions tested, irradiation with a 532 nm (green) LASER induced the most significant reduction in cell viability, as quantified in Figure 3. Data from the microscopy exclusion assay (Figure 3A) demonstrate a clear dose–response relationship; cell viability progressively decreased with increasing energy dose, reaching a minimum of 32.2% at 9.57 J/cm^2^. This effect was corroborated by the MTT metabolic viability assay (Figure 3B), which confirmed the high efficacy of the green LASER, showing a reduction in metabolic activity corresponding to a cell viability of approximately 40%.

It is crucial to highlight the dual specificity of this finding. First, neither blue nor red LASER irradiation managed to cause a significant reduction in viability. Second, the physical properties of the source emerged as a determining factor; green LED light, despite delivering the same energy doses, showed a marginal effect that was not statistically distinguishable from the LASER in the MTT assay (*p* = 0.24) and was significantly inferior in the microscopy count (*p* = 0.007). This implies that the coherence and/or high power density of the LASER are indispensable properties for effectively activating the cell death mechanism.

### 3.3. Irradiation Induces Profound Changes in the Spectroscopic Signature and Cell Morphology

**Figure 4:**
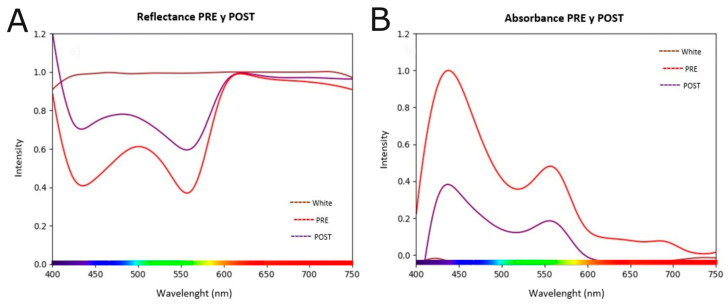
Reflectance (**A**) and absorbance (**B**) spectra of K-562 cells before (PRE) and after (POST) irradiation with a green LASER at 10 J/cm^2^. (**A**) shows an increase in reflectance after treatment, consistent with a lower photon capture by the decimated cell population. Complementarily and more significantly, (**B**) reveals a drastic decrease in the absorbance signal in the treated sample (POST, purple line) compared to the control (PRE, red line). This reduction, statistically significant (*p* = 0.001), serves as a direct biophysical signature of the loss of viable cells, corroborating the potent cytotoxic effect of the treatment.

The profound biological impact of the green LASER treatment was manifested in observable changes at both biophysical and morphological levels. The analysis of the cell population spectra before and after irradiation revealed a drastic alteration of their optical signature (Figure 4). An increase in reflectance was observed (Figure 4A) and, complementarily, a statistically very significant reduction (*p* = 0.001) in the absorbance signal across the entire visible spectrum (Figure 4B). This attenuation in absorbance is the direct spectroscopic manifestation of the loss of intact cells in the suspension.

**Figure 5:**
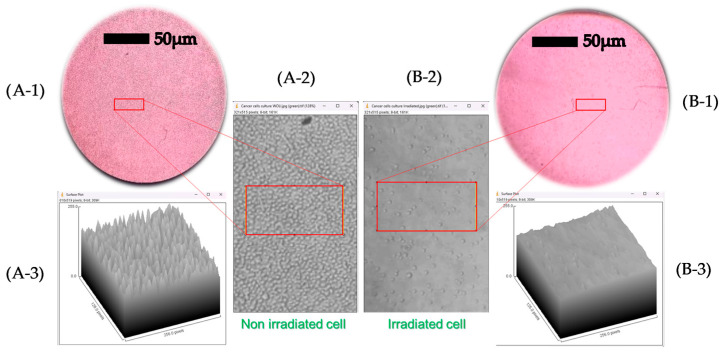
Phase-contrast micrographs (40X magnification) illustrating the morphological effect of green LASER irradiation on K-562 cells. (**A-1**) Control cell culture before irradiation (PRE), showing a high density of viable cells with their characteristic morphology. (**B-1**) The same culture field after irradiation (POST) with a green LASER at 10 J/cm^2^, where a drastic reduction in cell density is apparent. The adjacent insets magnify the central region of each field to emphasize the difference in confluency, providing clear visual evidence of the cytotoxicity induced by the treatment (**A-2**,**B-2**). Corroborating the results, a 3D surface plot is obtained showing a greater number of peaks in the area of the non-irradiated cells (**A-3**) than in that of the irradiated cells (**B-3**) (imagej.net). This reaffirms that the laser successfully induced controlled cell death in K562 cells.

These quantitative measurements were corroborated by direct visual evidence obtained through phase-contrast microscopy (Figure 5). Before irradiation, the cultures exhibited a high cell density characteristic of a non-optically treated population (Figure 5A). However, after exposure to the green LASER, the same region of the culture showed a marked decrease in cell density and the presence of debris, visually confirming the destructive nature of the treatment (Figure 5B).

Likewise, a comparison of the absorbance spectrum between the PRE and POST optical irradiation conditions (green laser, 10 J/cm^2^ dose) was carried out in the K-562 cell line, using the blank as a reference. The curves represent absorbance intensity (Y-axis) across the spectral range of 400 to 750 nm (X-axis). Visually, a substantial reduction in absorbance is observed after irradiation (POST curve) compared to the baseline condition (PRE curve), particularly in the spectrum region corresponding to 450–500 nm, where the maximum peak is located (~1.0 absorbance units in PRE vs. ~0.4 in POST), and in the secondary region between 520–550 nm (~1.0 absorbance units in PRE vs. ~0.4 in POST), suggesting significant alterations in cellular integrity.

**Figure 6:**
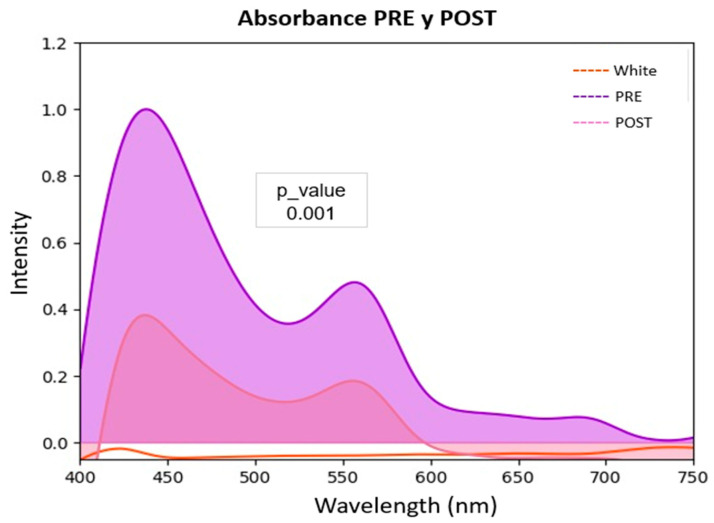
Area under the curve of the Absorbance spectrum PRE and POST-Green LASER IRRADIATION Dose 10 J/cm^2^ for calculation of POST live cells. The spectral comparison between the PRE and POST optical irradiation conditions (green laser, 10 J/cm^2^) shows significant reductions in absorbance after treatment, with maximum changes in the visible range between 450–500 nm and a second, smaller peak between 520–550 nm. The POST curve exhibits a smaller area under the curve (AUC), indicating cell death. This analysis was performed using OceanView software (version 2.0, Ocean Optics Inc., Largo, FL, USA) was used for spectral acquisition, and Python software (version 3.11, Python Software Foundation, Wilmington, DE, USA) was employed for post-processing and 3D visualization of optical data.

From a statistical standpoint, the null hypothesis (H_0_) was proposed that the absorbance distributions in the PRE and POST conditions are equal, against the alternative hypothesis (H_1_) which states there are differences between them. Given that the data correspond to measurements on the same sample under different conditions (PRE vs. POST) and normality was not assumed, the non-parametric Friedman test was applied, suitable for paired data in more than two measurements or in spectra discretized into multiple wavelengths. The analysis yielded a *p*-value = 0.001, below the α = 0.05 threshold, which allows rejecting H_0_ with a high level of confidence, concluding that the PRE and POST curves showed statistically significant differences (Figure 6). In terms of effect size, the observed discrepancy indicates not only a global difference but also a consistent spectral change, evidenced by a relative decrease in the area under the curve in the POST condition. This finding reinforces the hypothesis that optical irradiation with green light (10 J/cm^2^) caused a significant alteration in the cellular structure of the cell components.

The findings suggest that irradiation with a green laser at 10 J/cm^2^ caused a significant and localized change in the cellular optical properties, especially in the chromophores responsible for absorption in the 450–550 nm region. This pattern is associated with structural alterations affecting cellular integrity, which aligns with the decrease in cell viability observed in complementary analyses such as MTT cytotoxicity and cell biomass.

### 3.4. Cross-Verification Through Multiple Assays Confirms the Robustness of the Cytotoxic Effect

To ensure the validity of the results, the cytotoxicity induced by the most effective condition (green LASER at ~10 J/cm^2^) was quantified using three orthogonal methodologies. As summarized in Table 1, the data obtained showed remarkable consistency and convergence. The strong correlation between measurements of membrane integrity (Trypan Blue: 67.8%), mitochondrial activity (MTT: 59.8%), and cell biomass (Spectroscopy: 70.6%) provides conclusive and quantitatively solid evidence that irradiation with a 532 nm LASER induces a massive and reproducible lethal process in K-562 cells.

## 4. Discussion

The present study demonstrates, for the first time, that direct irradiation with 532 nm LASER light, in the absence of exogenous photosensitizers, induces massive, selective, and dose-dependent cytotoxicity in the K-562 CML cell line. This central finding, rigorously validated through three orthogonal quantification methodologies, opens a new avenue of research in the field of biophotonic therapies for hematological neoplasms. The following discussion will address the underlying mechanisms, contextualize these results within the existing literature, and explore the clinical and future implications of this innovative therapeutic approach.

### 4.1. Main Findings and Postulated Mechanisms

The most significant discovery of this work is the dual specificity of the cytotoxic effect: a strict dependence on both the wavelength (532 nm) and the physical properties of the light source (LASER vs. LED). The ineffectiveness of blue and red wavelengths, despite blue light being efficiently absorbed by the cells (Figure 2A), suggests that the effect is not a generic consequence of energy deposition but the result of activating a specific photochemical pathway. The absorption of light by K-562 cells in the green region (~560 nm) points to the existence of an endogenous chromophore that acts as the initiator of this lethal cascade. Although not directly identified, plausible candidates include components of the mitochondrial electron transport chain, such as cytochromes, or endogenous porphyrins, which are known to have absorption bands in this region of the spectrum (Soret and Q bands) and can act as efficient photosensitizers [19]. The activation of these molecules by 532 nm photons likely leads to the generation of reactive oxygen species (ROS), such as singlet oxygen, which trigger lethal oxidative stress, damaging critical organelles and activating programmed cell death or necrosis pathways [20].

Equally revealing is the drastic difference in efficacy between the LASER and the LED of the same wavelength. The superiority of the LASER implies that coherence and/or high instantaneous power density are indispensable properties for the observed effect. Unlike the non-coherent light from an LED, LASER radiation can induce non-linear effects or a more efficient saturation of the target chromophore’s excited states, thereby maximizing ROS production and subsequent cytotoxicity [12,21,22]. This finding underscores that the physical properties of light, not just the energy dose, are a critical parameter in the design of photonic therapies.

### 4.2. Comparison with Current Literature

These results are situated at a unique intersection between two fields of biophotonics: PDT and photobiomodulation (PBM). Conventional PDT is based on the administration of an exogenous photosensitizer drug that preferentially accumulates in tumor cells [23]. Our study fundamentally differs from this paradigm by achieving a cytotoxic effect without the need for any external agent, thus avoiding the pharmacokinetic complexities and systemic photosensitivity associated with classic PDT. While there are studies on the autofluorescence of leukemic cells, the exploitation of their endogenous chromophores to induce massive cell death with visible light is a scarcely explored field.

On the other hand, PBM uses low-intensity light, typically in the red and near-infrared spectrum, to stimulate cellular processes such as proliferation and tissue repair, often through the modulation of cytochrome c oxidase [24]. Our findings with green light at higher doses are on the opposite, phototoxic, end of the spectrum of cellular responses to light. This reinforces the concept of the “biphasic therapeutic window,” where wavelength and dose determine whether the cellular response is stimulatory or inhibitory, and demonstrates that green light, at an appropriate fluence, can be a potent inducer of cell death rather than a biostimulatory agent.

### 4.3. Clinical and Therapeutic Implications

The ability to selectively eliminate CML cells through direct irradiation opens promising therapeutic prospects, mainly in the context of ex vivo applications. The fundamental limitation of green light penetration into tissues makes a direct in vivo application unfeasible for a systemic disease like leukemia. However, this method is ideally suited for an ECP protocol. In such a procedure, the patient’s blood is circulated through an external device where leukocytes are exposed to 532 nm LASER irradiation before being reinfused [25]. This approach could serve as a non-pharmacological adjuvant therapy to reduce tumor burden, eliminate cells resistant to TKIs, or modulate the immune response, similar to how ECP is used in cutaneous T-cell lymphoma and graft-versus-host disease [26].

### 4.4. Study Limitations and Future Directions

While the findings of this in vitro proof-of-concept study are compelling, it is crucial to address its limitations and outline a clear path toward clinical translation. The immediate next step is to validate these results across a broader spectrum of CML cell lines and, most importantly, in primary cells obtained from CML patients to ensure the effect is not cell line-specific. However, the most critical step before considering any therapeutic application is to rigorously evaluate the selectivity of this method. Future experiments will focus on co-cultures and parallel irradiations of healthy hematopoietic cells, particularly peripheral blood mononuclear cells (PBMCs), to confirm that 532 nm LASER irradiation induces minimal toxicity in non-malignant cells. This is the cornerstone for its potential clinical use.

Concurrently, the underlying mechanism of cell death must be elucidated. While we postulate an ROS-mediated process, this needs to be confirmed by directly quantifying ROS generation post-irradiation and characterizing the specific cell death pathway (i.e., apoptosis versus necrosis) using assays such as flow cytometry with Annexin V/PI staining and caspase activity measurements. Positive outcomes from these essential validation and selectivity studies will pave the way for the next translational phase: the design and development of a prototype ECP device for ex vivo treatment, bringing this promising non-pharmacological approach one step closer to clinical application.

## 5. Conclusions

This study successfully established an in vitro proof-of-concept for a biophotonic protocol with therapeutic potential, fulfilling its objective of evaluating the effect of direct light irradiation on CML cells. The most significant finding is the unequivocal demonstration that irradiation with 532 nm LASER light, without the mediation of exogenous photosensitizers, induces potent and selective cytotoxicity in the K-562 cell line within a controlled laboratory setting. This effect was not only dependent on a specific wavelength but also on the physical properties of the light source, underscoring the superiority of coherent LASER radiation. These results lay the foundational groundwork for the development of a novel, non-pharmacological adjuvant therapeutic modality that could, following extensive further validation including selectivity against healthy cells, be adapted for ex vivo applications such as ECP for the treatment of CML.

## Figures and Tables

**Table 1:** Comparison of cell death percentages obtained by three quantification methods for irradiation with a green LASER at a dose of ~10 J/cm^2^.

Quantification Method	Light Source	Wavelength (nm)	Dose (J/cm^2^)	Cell Death (%)
Microscopy (Trypan Blue)	LASER	532	10	67.8
Spectroscopic (Absorbance)	LASER	532	10	70.6
Cytotoxicity Assay (MTT)	LASER	532	10	59.8

Abbreviations: MTT, colorimetric assay with 3-(4,5-dimethylthiazol-2-yl)-2,5-diphenyltetrazolium bromide. The data demonstrate high consistency among results obtained by methods that evaluate membrane integrity, metabolic activity, and cell biomass.

## Data Availability

Data is contained within the article.

## Abbreviations

The following abbreviations are used in this manuscript:

CML	Chronic Myeloid Leukemia
TKI	Tyrosine Kinase Inhibitor
DLS	Direct Light Stimulation
OTW	Optical Therapeutic Window
PDT	Photodynamic Therapy
ECP	Extracorporeal Photopheresis
ROS	Reactive Oxygen Species
SD	Standard Deviation
MTT	3-(4,5-dimethylthiazol-2-yl)-2,5-diphenyltetrazolium bromide assay
